# Dr. Samuel McCartney Bleakney

**Published:** 1912-03

**Authors:** 


DR. SAMUEL MCCARTNEY BLEAKNEY, University of Buffalo,1866, died at his home in Worthville, Pa., of pneumonia Feb. 2, 1912, aged 76.

